# Comparison of pancreatic respiratory motion management with three abdominal corsets for particle radiation therapy: Case study

**DOI:** 10.1002/acm2.12613

**Published:** 2019-05-23

**Authors:** Kai Dolde, Sergej Schneider, Sarah Stefanowicz, Merkur Alimusaj, Beate Flügel, Nami Saito, Esther G. C. Troost, Asja Pfaffenberger, Aswin L. Hoffmann

**Affiliations:** ^1^ Medical Physics in Radiation Oncology German Cancer Research Center (DKFZ) Heidelberg Germany; ^2^ National Center for Radiation Research in Oncology Heidelberg Institute for Radiooncology Heidelberg Germany; ^3^ Department of Physics and Astronomy University of Heidelberg Heidelberg Germany; ^4^ Institute of Radiooncology – OncoRay Helmholtz‐Zentrum Dresden‐Rossendorf Dresden Germany; ^5^ Faculty of Medicine and University Hospital Carl Gustav Carus, OncoRay – National Center for Radiation Research in Oncology Technische Universität Dresden, Helmholtz‐Zentrum Dresden‐Rossendorf Dresden Germany; ^6^ Center for Orthopedic and Trauma Surgery Heidelberg University Hospital Heidelberg Germany; ^7^ Department of Radiation Oncology University Clinic Heidelberg Heidelberg Germany; ^8^ Department of Radiotherapy and Radiation Oncology, Faculty of Medicine and University Hospital Carl Gustav Carus Technische Universität Dresden Dresden Germany; ^9^ German Cancer Consortium (DKTK) Partner Site Dresden, and German Cancer Research Center (DKFZ) Heidelberg Germany; ^10^ National Center for Tumor Diseases (NCT), Partner Site Dresden, Germany, German Cancer Research Center (DKFZ), Heidelberg, Germany; Faculty of Medicine and University Hospital Carl Gustav Carus, Technische Universität Dresden, and Helmholtz Association / Helmholtz-Zentrum Dresden-Rossendorf (HZDR), Dresden Germany

**Keywords:** 4D‐MRI, abdominal corset, image‐guided radiotherapy, motion management, pancreatic cancer, particle therapy

## Abstract

**Background and purpose:**

Abdominal organ motion seriously compromises the targeting accuracy for particle therapy in patients with pancreatic adenocarcinoma. This study compares three different abdominal corsets regarding their ability to reduce pancreatic motion and their potential usability in particle therapy.

**Materials and methods:**

A patient‐individualized polyurethane (PU), a semi‐individualized polyethylene (PE), and a patient‐individualized three‐dimensional‐scan based polyethylene (3D‐PE) corset were manufactured for one healthy volunteer. Time‐resolved volumetric four‐dimensional‐magnetic resonance imaging (4D‐MRI) and single‐slice two‐dimensional (2D) cine‐MRI scans were acquired on two consecutive days to compare free‐breathing motion patterns with and without corsets. The corset material properties, such as thickness variance, material homogeneity in Hounsfield units (HU) on computed tomography (CT) scans, and manufacturing features were compared. The water equivalent ratio (WER) of corset material samples was measured using a multi‐layer ionization chamber for proton energies of 150 and 200 MeV.

**Results:**

All corsets reduced the pancreatic motion on average by 9.6 mm in inferior–superior and by 3.2 mm in anterior‐posterior direction. With corset, the breathing frequency was approximately doubled and the day‐to‐day motion variations were reduced. The WER measurements showed an average value of 0.993 and 0.956 for the PE and 3DPE corset, respectively, and of 0.298 for the PU corset. The PE and 3DPE corsets showed a constant thickness of 2.8 ± 0.2 and 3.8 ± 0.2 mm, respectively and a homogeneous material composition with a standard deviation (SD) of 31 and 32 HU, respectively. The PU corset showed a variable thickness of 4.2 − 25.6 mm and a heterogeneous structure with air inclusions with an SD of 113 HU.

**Conclusion:**

Abdominal corsets may be effective devices to reduce pancreatic motion. For particle therapy, PE‐based corsets are preferred over PU‐based corset due to their material homogeneity and constant thickness.

## 1 INTRODUCTION

Particle therapy (PT) with protons or carbon ions is increasingly becoming an alternative treatment modality for conventional photon‐based radiotherapy (XT) in thoracic and abdominal tumours. This is mainly due to their ability to deposit almost all their dose at the end of their track (the so‐called “Bragg peak”), thereby minimizing the dose to organs at risk both in the initial beam path and beyond the Bragg peak.^1^ Appropriate immobilisation of the target volume is a key component in the treatment process for precise dose delivery. This is particular important for PT due to its higher sensitivity to density variations caused by inter‐ and intra‐fractional changes in patient anatomy. Morphological changes along the beam path due to organ motion, deformation and organ filling can influence the position of the Bragg peak relative to the target volume. Furthermore, the use of active dose delivery techniques that employ scanned beams to volumetrically scan a mono‐energetic Bragg peak over the target volume in combination with intra‐fractional organ motion may further degrade precise dose delivery due to the interplay effect.^2^, ^3^, ^4^, ^5^, ^6^ This may result in over‐ or underdosage of the target volume and additional unwanted dose deposition in adjacent organs at risk.

For thoracic and abdominal organs, the main source of motion is respiration. To reduce respiration‐induced uncertainties in PT, breathing motion can be accounted for by 4D treatment planning and optimizations,^3^, ^6^, ^7^, ^8^, ^9^, ^10^, ^11^ tumour tracking,^12^, ^13^ gating scenarios,^14^, ^15^, ^16^, ^17^ or by physically reducing respiratory‐induced motion by abdominal compression techniques.^18^, ^19^, ^20^ For PT of targets in the upper gastro‐intestinal tract, including the liver and pancreas, motion mitigation by means of abdominal compression bands or pressure plates can, however, exacerbate the range uncertainties due to a poor setup reproducibility and consequential edge effects of the devices used.^21^ In particular, pressure plates only allow for limited usability due to their bulky setup.

As a solution to reduce breathing induced tumour motion in patients with pancreatic cancer, polyurethane‐based customized abdominal corsets have recently been used in stereotactic XT.^18^ Studies using magnetic resonance imaging (MRI) in multi‐planar 2D‐cine or 4D mode have shown the ability of abdominal corsets to reduce pancreatic motion mainly in inferior‐superior direction.^18^, ^19^, ^20^

However, the corsets used in XT may not be applicable for PT, since for the latter, the reproducibility of the setup for immobilization devices placed in the beam path and an exact knowledge of their material properties (i.e. thickness and homogeneity) is critical to calculate the beam penumbra and range in the patient. When placed in the beam path, immobilization devices modify the position of the Bragg peak, and therefore it is crucial to understand how the dose distribution is affected.^21^ In PT, the water equivalent ratio (WER) of any material placed in the beam path needs to be known to ensure that the treatment planning system is able to accurately take the effects on beam penumbra and range into account.

Furthermore, it is unknown to which level of customization the corsets need to be manufactured in order to achieve an adequate level of motion reduction, while a reproducible positioning is still ensured. This needs to be investigated before corsets can be clinically used for PT.

Therefore, in this study we compared three types of abdominal corsets that differ in terms of material composition, thickness, homogeneity, size, and degree of patient customization. The goal of this study was to determine which corsets are suitable for PT regarding their motion reduction capabilities and their material properties.

## 2 MATERIALS AND METHODS

### 2.1 Abdominal corsets

**Figure 1:**
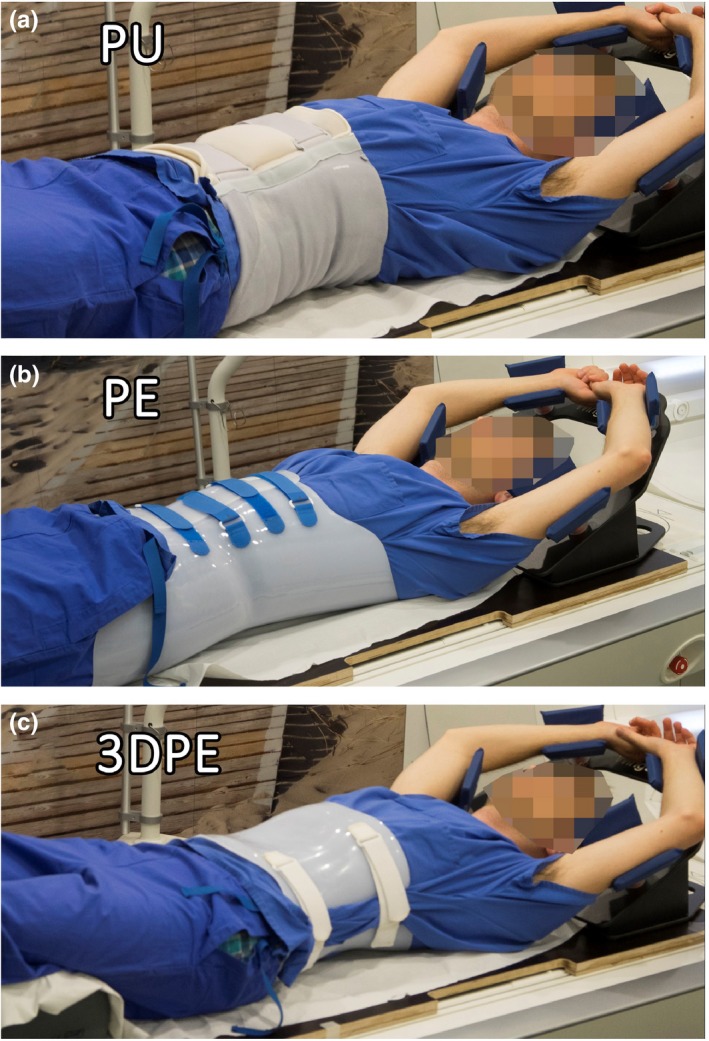
Healthy subject wearing the three different corsets evaluated in this study: A patient‐individualized polyurethane corset (PU), a semi‐specific polyethylene corset (PE) and a patient‐individualized three‐dimensional‐surface scan‐based polyethylene corset (3DPE)

Three different types of abdominal corsets were evaluated: a custom‐made solid foam‐based polyurethane corset (PU), a prefabricated polyethylene corset (PE) and a custom‐made 3D‐scan based polyethylene corset (3DPE), see Fig. 1.

#### 2.1.1 Polyurethane corset (PU)

The patient‐individualized PU corset (OKMSystem®, OKM Química Ortopédica, Girona, Spain) is a custom‐made (Orthopädie‐ und Rehatechnik Dresden GmbH, Dresden, Germany) solid foam‐based corset enclosed in a cotton fabric, which is moulded in soft condition directly around the lumbar spine, where it hardens within 20 min after mixing its initial liquid components isocyanate and polyol. The corset is fastened using hook‐and‐pile fasteners, which were positioned ventrally, 5 cm away from the mid‐sagittal plane. In inferior‐superior direction, the corset ranged from the ileum to the xiphoid process, compressing the stomach and lower four ribs. Its manufacturing and solidifying require approx. 30 min.

#### 2.1.2 Polyethylene corset (PE)

This Boston Overlap Brace (BOB)‐hull orthoses (Basko Healthcare, Hamburg, Germany) corset was fitted in cooperation with the Prosthetics and Orthotics Department of the Heidelberg University Hospital, Germany. It consists of a polyethylene (PE) prefabricated symmetrically formed module that enables individual adaptation based on size and gender (i.e. male and female shapes). Three different lordosis angles (0°/15°/30°) are available. Trim lines are adjusted to the patient's individual anatomy to avoid uncomfortable pressure to the greater trochanter and the axilla. The corset is ventrally closed by four hook‐and‐pile fasteners, such that no fastener material or buckles are present at the side and back of the subject. This type of corset allows for an adequate stabilization of the lumbosacral spinal segments from the sacral spine (S1) up to the thoracic spine (Th8). Corset adjustment lasts for approximately 30 min.

#### 2.1.3 3D‐scanned polyethylene corset (3DPE)

The patient‐individualized 3DPE corset (Orthopädie‐ und Rehatechnik Dresden GmbH, Dresden, Germany) is custom‐made based on an optical 3D‐surface scan (Artec Eva®, Artec3D, Luxembourg, Luxemburg) of the patient. The 3D‐surface scan is performed in treatment position using a vacuum cushion (TapMed Medizintechnik Handels GmbH, Habichtswald, Germany) for modelling the dorsum from its imprint. The surface model is finally replicated as a milled wooden model onto which a 5 mm PE plate (Streifylast®/Trolen) is thermoformed in an industrial oven at 150°C. The individually shaped corset is finally adjusted and equipped with hook‐and‐pile fasteners, which were positioned on the anterior and posterior left side of the corset 5 and 12 cm away from the mid‐sagittal plane, respectively. In inferior‐superior direction, the corset ranged from the sternum to the ilium, compressing the stomach and the lower five ribs. The modelling and manufacturing requires two 20 min outpatient clinic appointments and a total manufacturing time of 3 days.

### 2.2 Material analysis

#### 2.2.1 Homogeneity and thickness

From all three corsets, computed tomography (CT) scans were acquired (Somatom Definition AS, Siemens Healthineers, Erlangen, Germany) to assess material homogeneity and variations of thickness (pixel size 0.8 × 0.8 mm^2^, slice thickness 2 mm, tube voltage 140 kVp, tube current‐time product 80 mAs). To quantify the size and number of air inclusions found in the PU corset, the inclusions were automatically segmented based on a density threshold in the CT image and analysed by means of a connected‐components analysis using MATLAB (MATLAB R2017b, The Mathworks Inc., Natick, MA). The corset thickness was measured as the full width at half maximum (FWHM) at five different positions in five different slices in the CT images, using the open‐source software ImageJ. Due to the varying thickness of the PU corset, the maximum and minimum thickness were determined in treatment‐relevant regions, i.e. at beam angles between 150°−220°, since pancreatic cancer patients are treated with two or three posterior oblique beams at our PT facilities in Dresden and Heidelberg.

#### 2.2.2 Water equivalent ratio

The WER is defined as the ratio of the mass thickness of water and the given material (in g/cm^2^) that leads to the same beam energy loss.^22^ WER measurements were performed at OncoRay (Dresden, Germany) at two different proton energies (150, 200 MeV) with a high‐resolution multi‐layer ionization chamber (Giraffe, IBA Dosimetry, Schwarzenbruck, Germany) that measured the shift of the single Bragg peak along the central beam axis after penetrating the respective corset samples. The proton range was determined by the depth of the 80% dose level at the distal dose fall‐off of the Bragg peak (*R*_80_). The two samples of the PU (PU1 and PU2, respectively) and PE corsets were cut out of an already manufactured corset, while the 3DPE sample was provided from raw material before its thermoforming. The thickness of the samples was measured with a calliper at three different spots in the central region of the respective sample.

### 2.3 MRI acquisition and motion analysis

To quantify respiration‐induced pancreatic motion, a healthy male volunteer was scanned in treatment position with the arms positioned superior to the head (WingSTEP, Innovative Technologie Völp, Innsbruck, Austria) on a custom‐made wooden flat‐table top overlay inside a 1.5 T MR scanner (Magnetom Aera, Siemens Healthineers) using a 32‐channel phased array torso coil, which was placed directly on the corset/volunteer. The volunteer underwent time‐resolved volumetric 4D‐MRI and fast single‐slice 2D cine‐MR imaging while subsequently wearing one of the three corsets and without corset for reference. The scans were performed twice on two consecutive days to investigate reproducibility of the pancreatic motion reduction. No specific breathing instructions were given to the volunteer aiming to acquire relaxed free‐breathing patterns.

For the 4D‐MRI measurements, a T_1_‐weighted gradient echo sequence with radial golden angle stack‐of‐stars sampling was used to acquire 3D images under free breathing of the subject (field of view = 384 × 384 mm^2^, pixel spacing = 1.5 × 1.5 mm^2^, slice thickness = 3 mm, spokes per partition = 2100, bandwidth per pixel = 610 Hz, echo time (TE) = 1.5 ms, repetition time (TR) = 3.3 ms, flip angle (α) = 12°, acquisition time = 8 min). The raw data were reconstructed offline, using a motion‐compensated iterative reconstruction algorithm, based on a *k*‐space‐centre self‐gating signal, which provides up to 20 overlapping breathing phases.^23^ On each 4D‐MRI data set, the pancreas was manually delineated on both the end‐inhale and end‐exhale images using the open‐source software MITK and validated by an experienced radiation oncologist. The centre‐of‐mass (COM) of these binary delineations was determined to estimate pancreatic motion in inferior–superior (IS), anterior–posterior (AP) and left–right lateral (LR) directions.

To allow an analysis of potential changes in breathing behaviour, single‐slice 2D cine‐MRI were acquired using a TrueFISP sequence in coronal orientation with 200 measurements at a frame rate of approximately 4 Hz (field of view = 384 × 384 mm^2^, pixel spacing = 1.5 × 1.5 mm^2^, slice thickness = 4 mm, bandwidth per pixel = 1030 Hz, TE = 1.26 ms, TR = 222.3 ms, α = 55°). The coronal cine‐MR images were used to evaluate the diaphragm position during individual breathing cycles. The diaphragm position was tracked automatically by means of an in‐house developed MATLAB‐based algorithm, which determines the position of the largest gradient in image intensity, i.e. at the interface of liver and lung. The 2D cine‐MRI data was used to determine the real‐time motion amplitudes in IS direction as well as the length of breathing cycles, whereas the 4D‐MRI data provides information averaged over several breathing cycles in IS, LR and AP directions.

### 2.4 Clinical implementation

The clinical implementation of the 3DPE corset in PT of abdominal cancer patients and the integration of such corsets into the treatment planning was evaluated in an in‐house ongoing clinical study with 9 patients.

## 3 RESULTS

### 3.1 Material analysis

#### 3.1.1 Homogeneity and thickness

**Figure 2:**
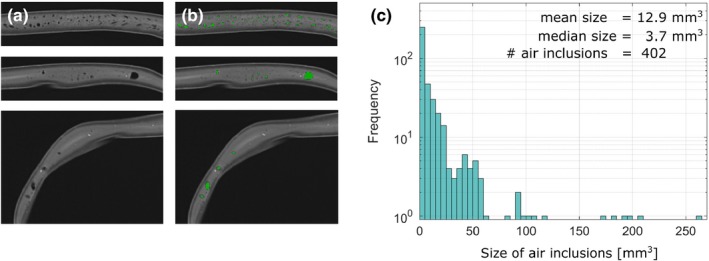
The computed tomography scan of the polyurethane corset corset shows air inclusions of different volumes in three transversal slices (a) and superimposed threshold‐segmented volumes in green (b). The distribution of the air inclusion volumes (c) shows 95% to have a volume of < 50 mm^3^ with maximum volumes of up to 260 mm^3^.

All measured material properties are summarized in Table 1. The CT scan confirmed a homogeneous material in the PE and 3DPE corsets with average ± standard deviation CT values of −130 ± 31 and −107 ± 32 HU, respectively as well as a constant thickness of 2.8 ± 0.2 and 3.8 ± 0.2 mm, respectively. The PU corset was inhomogeneous comprising air inclusions throughout the whole corset with sizes of up to 7.6 mm in diameter and a mean volume of 12.9 mm^3^ (range 1.2–263.7 mm^3^). 95% of the air inclusions were smaller than 50 mm^3^, and 5% had larger volumes of up to 263.7 mm^3^ (Fig. 2). Moreover, smaller hyperintensities were observed in the CT images. The HU density of these small hyperintensity volumes was not corresponding to components in the list of materials from the manufacturer and the origin of these hyperintensities was not identified. For the PU corset, the average CT value was −677 ± 113 HU with values ranging from −1024 HU (air) to + 990 HU (hyperintensities). Furthermore, its thickness varied between 4.2–25.6 mm.

**Table 1:** Material analysis of the three corsets. The measured thickness of corsets and samples, material homogeneity as well as the average measured water equivalent ratio (WER) for protons at energies of 150 and 200 MeV, respectively, are listed. For corset thickness, sample thickness and material homogeneity, the indicated uncertainties represent the standard deviation of the measured value in multiple measurements (*N* ≥ 15). The uncertainties of the WER measurement resulted from the uncertainty of the underlying Bortfeld‐Fit of the depth‐dose curve.

Corset	Corset thickness (mm)	Sample thickness (mm)	Material homogeneity (HU)	Proton beam energy (MeV)	WER
PE	2.8 ± 0.2	2.53 ± 0.07	−130 ± 31	150	0.956 ± 0.002
				200	0.956 ± 0.002
3DPE	3.8 ± 0.2	4.87 ± 0.03	−107 ± 32	150	0.988 ± 0.002
				200	0.993 ± 0.002
PU1	4.2 − 27.7	11.42 ± 0.34	−677 ± 113	150	0.297 ± 0.002
				200	0.297 ± 0.002
PU2	4.2 − 27.7	10.10 ± 0.29	−677 ± 113	150	0.298 ± 0.002
				200	0.298 ± 0.002

Abbreviations: PE = polyethylene, 3DPE = 3D‐scanned polyethylene, PU = polyurethane.

#### 3.1.2 Water equivalent ratio (WER)

The average thickness of the PE and 3DPE samples used for the WER measurements was 2.53 ± 0.07 and 4.87 ± 0.03 mm, respectively. The average thickness of the PU1 and PU2 samples was 11.42 ± 0.34 and 10.10 ± 0.29 mm, respectively. The WER of the respective corsets showed values between 0.297 and 0.993. The detailed values are listed in Table 1.

### 3.2 Motion reduction

**Figure 3:**
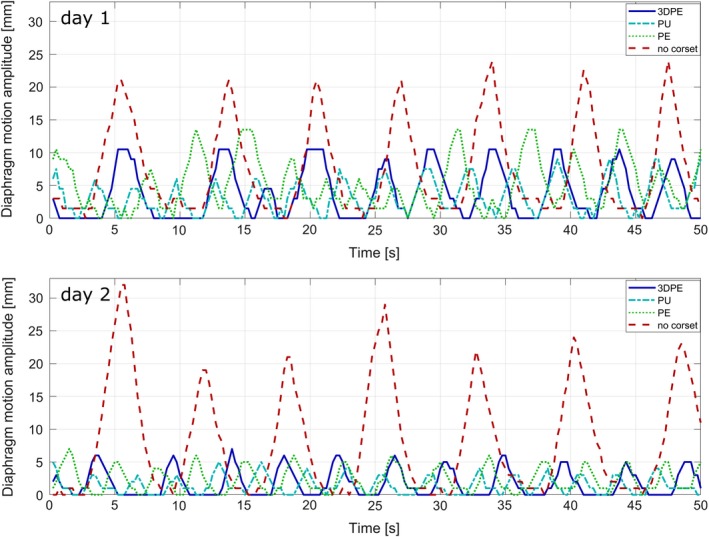
Example of diaphragm motion amplitudes in inferior‐superior direction without and with corsets measured by two‐dimensional‐cine MRI in the healthy volunteer on two consecutive days. *Abbreviations*. 3DPE = 3D‐scanned polyethylene, PU = polyurethane, PE = polyethylene

Without corset, the analysis of the 2D cine‐MRI showed an average diaphragm motion in IS direction of 23.1 mm (range 19.0–32.0 mm). With the corsets, this motion was significantly reduced to an average of 5.3 mm (range 3.0–9.0 mm), 7.1 mm (range 3.0–13.5 mm), and 7.4 mm (range 3.0–10.5 mm) for the PU, PE and 3DPE corset, respectively. Hence, on average, the diaphragm motion amplitude in IS direction was reduced by 77%, 69% and 66% with the PU, PE and 3DPE corset, respectively. When wearing either of the corsets, the breathing frequency increased, leading to a mean length of breathing cycles of 3.2 ± 0.4, 3.3 ± 0.6, and 4.6 ± 1.1 s for the PU, PE and 3DPE corset, respectively instead of 7.1 ± 0.7 s without corset. Examples of diaphragm motion patterns without and with the corsets as measured by 2D cine‐MRI for both days are illustrated in Fig. 3. It is also visible, that the end‐exhale phase (i.e. the baseline in Fig. 3) is stable and reproducible, while the peak positions in the end‐inhale phases may vary over the breathing cycles. In particular without corset, variations in the motion amplitudes of up to 13 mm were observed within one minute, compared to maximum variations of 7.5 mm with corset.

**Figure 4:**
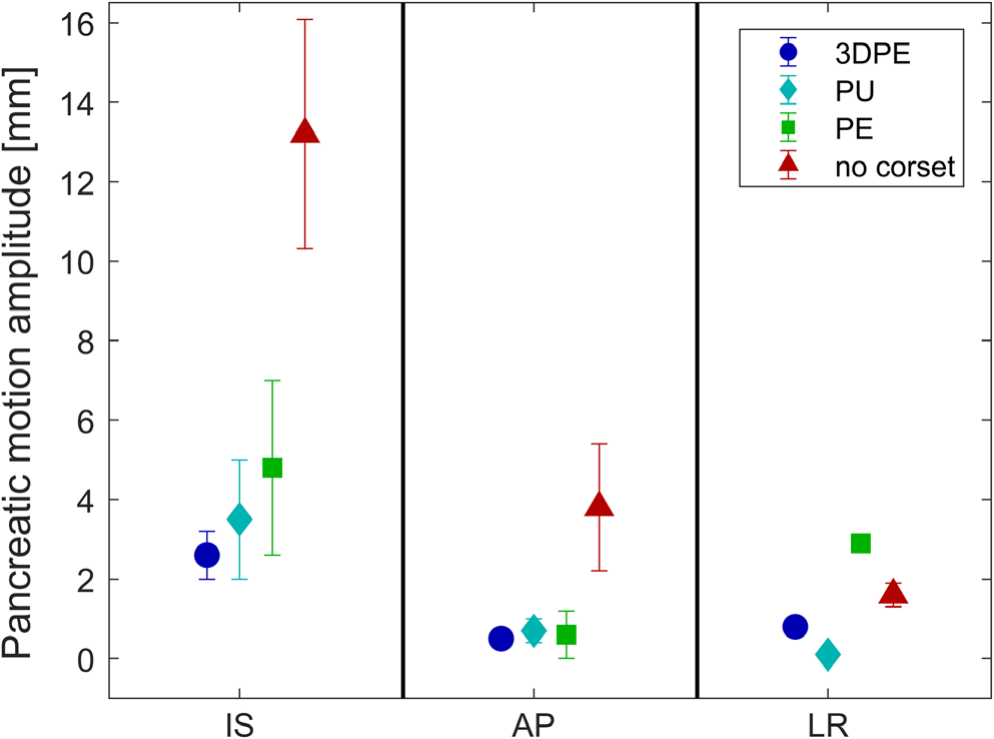
Average centre‐of‐mass motion of the pancreas in inferior–superior (IS), anterior–posterior (AP) and left–right (LR) direction with the PU, PE and 3DPE corsets and without corset. The error bars represent the range of motion on the two consecutive days on which the measurements were performed. *Abbreviations*: 3DPE = three‐dimensional‐scanned polyethylene, PU = polyurethane, PE = polyethylene

**Figure 5:**
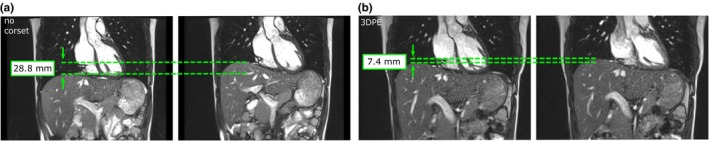
Coronal two‐dimensional cine‐magnetic resonance imaging scans in the end‐inhale (left) and end‐exhale (right) breathing phase (a) without corset and (b) with the three‐dimensional‐scanned polyethylene corset. The green dashed lines illustrate the amplitude of diaphragm motion

**Figure 6:**
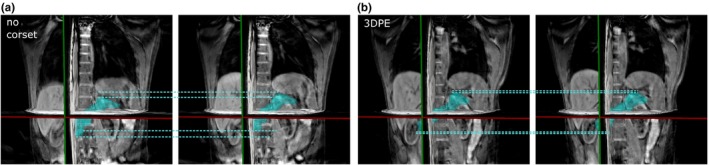
Illustration of four‐dimensional‐magnetic resonance imaging scans with respective pancreas delineations in the end‐inhale (left) and end‐exhale (right) breathing phase (a) without corset and (b) with the three‐dimensional‐scanned polyethylene corset. The motion range of the most inferior/superior part of the pancreas in IS direction is illustrated by blue dashed lines

4D‐MRI showed that all three corsets reduced pancreatic COM motion in both IS and AP direction. For all corsets, the largest absolute motion reduction was found in the IS direction, measuring on average 2.6–8.8 mm with corset as opposed to 13.2 mm without corset. In AP direction, the mean amplitude was reduced from 3.8 mm without corset to 0.5–0.7 mm with corset. In LR direction, mean amplitudes without corset of 1.6 mm were observed, which were decreased for the PU and 3DPE corset to 0.1–0.7 mm and increased by the PE corset to 2.9 mm. The detailed values are illustrated in Fig. 4. Figures 5 and 6 show motion amplitudes of 2D cine‐MRI and 4D‐MRI with and without the 3DPE corset, respectively.

### 3.3 Clinical implementation

**Figure 7:**
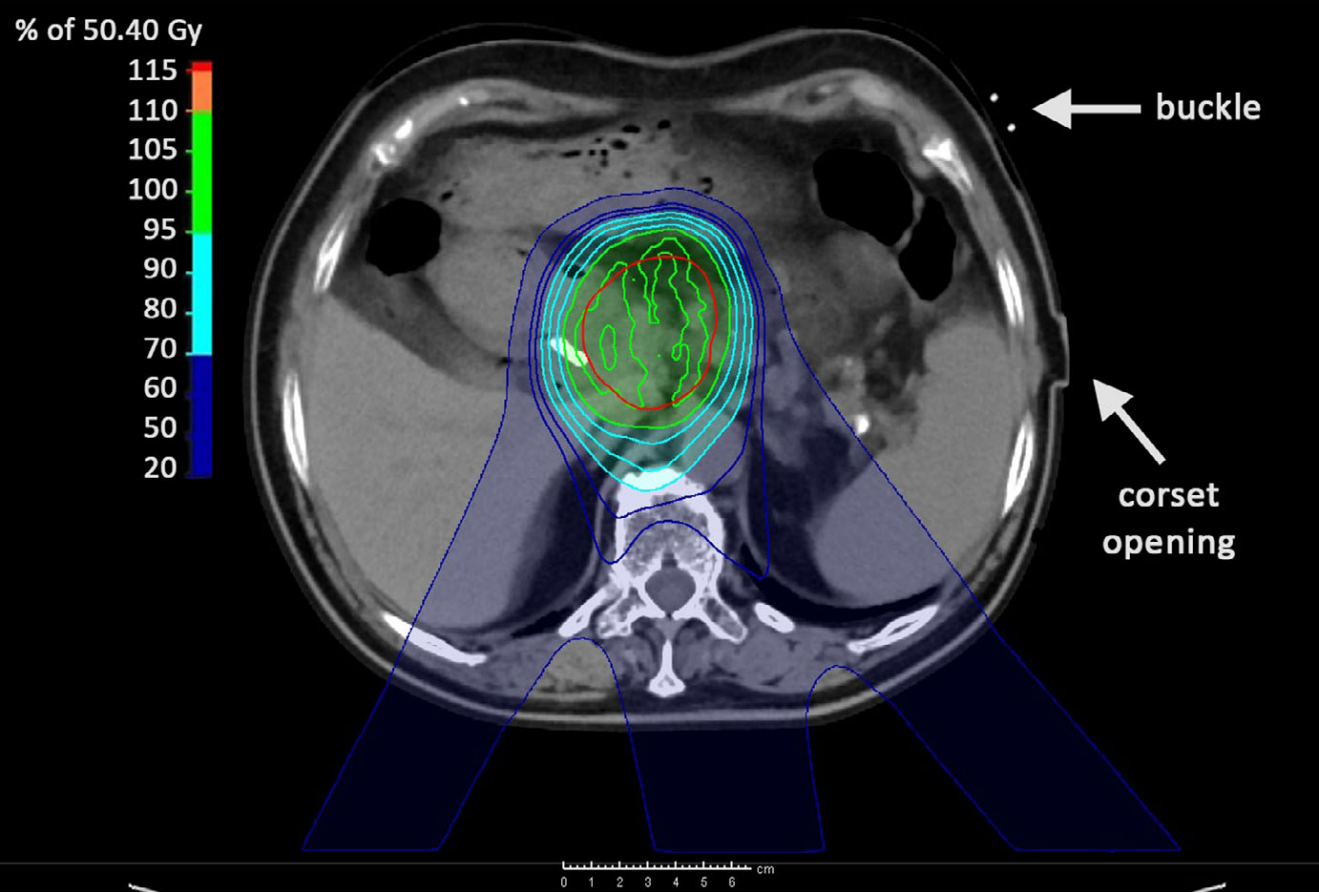
Example of an applied robust optimized pencil beam scanned proton therapy plan of a patient with carcinoma of the pancreatic head wearing the three‐dimensional‐scanned polyethylene corset. Three beam angles were applied (140°, 170°, 208°) to accumulate a dose of 50.4 Gy within 28 fractions in the internal clinical target volume (red contour). The buckle of the corsets hook‐and‐pile fasteners and the corset opening are marked. Beam angles penetrating these structures (40°–100°) were avoided.

Our in‐house ongoing clinical study with 9 patients showed that it was sufficient to directly transform the measured CT values of the planning CT to WER values with the clinically used Hounsfield look‐up table (HLUT). This was due to the adipose tissue equivalency^24^ of both CT values (−107 ± 32 HU) and WER values in proton irradiation (0.991 ± 0.002) in combination with the small corset thickness of 3.8 mm. An already conservatively estimated error of 10% in the translation of HU to the stopping power would consequently only lead to a maximum error of 0.38 mm in the proton range prediction. In Fig. 7, a clinically applied PT plan is shown, in which a patient with an adenocarcinoma of the pancreatic head was irradiated with three proton beams with angles between 140° and 208° to deposit a dose of 50.4 Gy within 28 fractions to the internal clinical target volume (iCTV). As illustrated in Fig. 7, the corset was designed such that both the buckles of the hook‐and‐pile fasteners as well as the edges of the corset opening were placed on the patient's left lateral side and thus they do not interfere with the applied PT beams. With this design, a potential positional variability of the buckles and opening could be ignored during RT.

## 4 DISCUSSION

All three corsets evaluated in this study reduced pancreatic motion, in particular in IS direction. Compared to the study by Heerkens et al.^18^, investigating the motion reduction by use of a PU corset and reporting a mean IS motion reduction of 4 mm with a large inter‐patient variability, we observed a mean pancreatic motion reduction of around 8 mm in IS direction in a single subject. We additionally observed an AP motion reduction of 3 mm, which was not the case in the previous study.^18^ However, as reported in previous corset studies,^18^, ^19^, ^20^ such results are highly patient‐specific. Therefore, from our study no general motion reduction magnitudes can be deduced.

Moreover, as opposed to our corset design, covering the ribs and abdomen in order to reduce both abdominal respiratory motion and chest breathing, the corset used by Heerkens et al.^18^ left out the ribs to promote chest breathing. We therefore hypothesize that these different corset designs may contribute to different observed motion reduction patterns.

While Heerkens et al.^18^ planned to use the corset for stereotactic XT, we set the focus on its applicability for PT, for which corset thickness and homogeneity are more important than for XT. With regard to the material properties, both the PE and the 3DPE corset show potential to be used in particle therapy since they have a constant thickness and are made of homogeneous material. From a manufacturing point of view, the 3DPE corset is more time‐consuming and costly as a consequence of its more patient‐specific manufacturing procedure. This, on the other hand, allows for flexibility in the design and hence a positioning of corset opening and buckles, which would avoid beam edge effects. The design of the 3DPE corset considered that beam angles between 40° and 100° were *a priori* not intended for pencil beam scanned PT planning since the presence of the stomach and the large intestine prevents the usage of left lateral (oblique) beam angles for pancreatic cancer patients.^25^, ^26^ Hence, in our study, the left lateral part was chosen as an opening. The PE corset, on the other hand, shows partially overlapping material layers at the anterior side, where the four hook‐and‐pile fasteners are placed (see Fig. 1). Since for this corset the opening is fixed by design, it compromises the use of anterior beams, since edge effects may occur due to the fact that the beam will pass across edges of the corset layers or the closing buckles. Although the treatment planning system will take the resulting range shifts into account, small shifts in patient position relative to the edges will impact the range of the traversing beam. In order to minimize the impact of air gaps on beam penumbra, the corset should closely match the patients' skin surface. However, since no CT scan of the healthy volunteer wearing the corset could be acquired, the fitting accuracy of the corset to the body surface could not be investigated.

The WER measurements show that both the PE corsets with their WER of 0.956─0.991 as well as the PU corset with a WER of 0.298 would have an impact on the range shifts in PT, and hence need to be included in the treatment planning. The measured WER showed to be constant for the two proton beam energies used, which is in good agreement with literature.^27^, ^28^ The PU corset showed pronounced wrinkles, air cavities and a variable thickness. Depending on the size of air cavity in the beam path, this could lead to range shifts from 1.3–7.6 mm for varying corset thicknesses of 4.2–25.6 mm. For XT, these drawbacks would have no detrimental effect on dosimetry, leading to a patient‐individualized solution, which has low manufacturing costs and lead time. However, for XT purposes, an increased skin dose by the corset needs to be considered.^18^ For PT, the air inclusions in the PU corset make it unsuitable, since the resulting range shifts lead to increased range uncertainties.^29^

The difference in measured thickness between the PE‐based sample used for WER determination and the corsets themselves is partially influenced by the different measurement procedures. While the thickness of the samples was measured with a calliper, it was determined as FWHM values in the respective CT scans for the corset. Due to the pixel width of 0.8 mm in the CT scans, the determination of the small corset thickness may be distorted. Furthermore, the discrepancy of the measured thickness of the 3DPE corset and the samples used for WER measurements results from the thermoforming process underlying the corsets manufacturing. This leads to a decreased thickness of the thermoformed corset. For fractionated radiation treatment of patients with pancreatic carcinoma, it is well‐known that intra‐abdominal anatomy changes occur from day to day, depending on the filling of the stomach and bowel.^30^ Moreover, substantial weight loss has been observed in these patients during the course of treatment.^31^ These factors compromise the setup reproducibility and may require the corset pressure to be adjusted between treatment fractions in order to secure adequate immobilization. This study has shown that pancreatic motion reduction obtained with patient‐individualized corsets is similar to that of the semi‐specific corset. Therefore, semi‐individualized corsets may be sufficient, which would have the advantages of reusability and short manufacturing time. However, substantial inter‐patient variability can be expected. This variability may depend on natural breathing patterns (i.e., chest or abdominal breathing), the size and location of the primary tumour, and its infiltration into surrounding tissues. Therefore, a comparative study in a large patient cohort is mandatory to get good statistics and analyse subgroups.

From a logistic point of view, the construction and adjustment of the PE and 3DPE corsets requires the cooperation with an orthopaedic institution and the technical infrastructure. Conversely, the clinical staff can produce the PU corset, after having undergone an initial training. While the PU and non‐individualized PE corsets required only 30 min of adjustment time, for the 3DPE‐corset a manufacturing time of 3 days was needed, which also translates to the differing costs of these corsets.

How to select patients eligible for wearing a corset during the course of radiation therapy is an open question. A previous study with liver tumour patients reported on dosimetric benefits of abdominal compression using a pressure belt for moderate and large tumour motion amplitudes.^32^ For patients with initially small tumour motion of <7 mm, however, abdominal compression was found to be needless. Furthermore, the selection of suitable patients may depend on the tumour infiltration, the patients' condition and willingness to tolerate the abdominal pressure during repeated imaging and irradiation fractions. However, preliminary results of an in‐house on‐going clinical trial with currently 9 patients included, show that a customized abdominal corset is well‐tolerated by patients with tumours in the upper abdomen (e.g., pancreas, liver, gall bladder) undergoing PT.

## 5 CONCLUSION

All three abdominal corsets were found to reduce breathing‐induced pancreatic motion to a comparable degree in this case study, in particular in inferior‐superior direction. Conclusions derived from this case study should be confirmed by a larger study with patients. Due to their well‐defined thickness and material homogeneity, as well as the favourable water equivalent ratios, the polyethylene corsets are suitable candidates for abdominal compression in particle therapy.

## CONFLICT OF INTEREST

No conflicts of interest.

## CONSENT FOR PUBLICATION

The patient provided written informed consent to publish treatment‐related data in an anonymised manner.

## References

[1] Durante M , Loeffler JS . Charged particles in radiation oncology. Nat Rev Clin Oncol. 2010;7:37–43. 19949433 10.1038/nrclinonc.2009.183

[2] Phillips MH , Pedroni E , Blattmann H , Boehringer T , Coray A , Scheib S . Effects of respiratory motion on dose uniformity with a charged particle scanning method. Phys Med Biol. 1992;37:223–233. 1311106 10.1088/0031-9155/37/1/016

[3] Bert C , Grözinger SO , Rietzel E . Quantification of interplay effects of scanned particle beams and moving targets. Phys Med Biol. 2008;53:2253–2265. 18401063 10.1088/0031-9155/53/9/003

[4] Seco J , Robertson D , Trofimov A , Paganetti H . Breathing interplay effects during proton beam scanning: simulation and statistical analysis. Phys Med Biol. 2009;54:283–294. 10.1088/0031-9155/54/14/N01 19550002

[5] Knopf A , Hong TS , Lomax X . Scanned proton radiotherapy for mobile targets‐the effectiveness of re‐scanning in the context of different treatment planning approaches and for different motion characteristics. Phys Med Biol. 2001;56:7257–7271. 10.1088/0031-9155/56/22/016 22037710

[6] Dolde K , Naumann P , Dávid C , et al. 4D dose calculation for pencil beam scanning proton therapy of pancreatic cancer using repeated 4DMRI datasets. Phys Med Biol. 2018;63:165005. 30020079 10.1088/1361-6560/aad43f

[7] Dolde K , Zhang Y , Chaudhri N , et al. 4DMRI‐based investigation on the interplay effect for pencil beam scanning proton therapy of pancreatic cancer patients. Radiat Oncol. 2019;14:30. 30732657 10.1186/s13014-019-1231-2 PMC6367829

[8] Batista V , Richter D , Chaudhri N , Naumann P , Herfath K , Jäkel O . Significance of intra‐fractional motion for pancreatic patients treated with charged particles. Radiat Oncol. 2018;13:120. 29941049 10.1186/s13014-018-1060-8 PMC6020245

[9] Kraus KM , Heath E , Oelfke U . Dosimetric consequences of tumour motion due to respiration for a scanned proton beam. Phys Med Biol. 2011;56:6563–6581. 21937770 10.1088/0031-9155/56/20/003

[10] Bert C , Durante M . Motion in radiotherapy: particle therapy. Phys Med Biol. 2011;56:R113–R144. 21775795 10.1088/0031-9155/56/16/R01

[11] Graeff C . Motion mitigation in scanned ion beam therapy through 4D‐optimization. Physica Med. 2014;30:570–577. 10.1016/j.ejmp.2014.03.011 24818997

[12] Bert C , Saito N , Schmidt A , Chaudhri N , Schardt D , Rietzel E . Target motion tracking with a scanned particle beam. Med Phys. 2007;34:4768–4771. 18196804 10.1118/1.2815934

[13] Zhang Y , Knopf A , Tanner C , Lomax AJ . Online image guided tumour tracking with scanned proton beams: a comprehensive simulation study. Phys Med Biol. 2014;59:7793–7817. 25420088 10.1088/0031-9155/59/24/7793

[14] Mori S , Yanagi T , Hara R , et al. Comparison of respiratory‐gated and respiratory‐ungated planning in scattered carbon ion beam treatment of the pancreas using 4D computed tomography. Int J Radiat Oncol Biol Phys. 2010;76:303–312. 19733015 10.1016/j.ijrobp.2009.05.026

[15] Miki K , Shinichiro M , Miho S , Shigeru Y . Gated carbon‐ion scanning treatment for pancreatic tumour with field specific target volume and organs at risk. Physica Med. 2016;32:1521–1528. 10.1016/j.ejmp.2016.11.009 27884463

[16] Pepin EW , Wu H , Shirato H . Dynamic gating window for compensation of baseline shift in respiratory‐gated radiation therapy. Med Phys. 2011;38:1912–1918. 21626924 10.1118/1.3556588 PMC3069997

[17] Zhang Y , Huth I , Weber DC , Lomax AJ . A statistical comparison of motion mitigation performances and robustness of various pencil beam scanned proton systems for liver tumour treatments. Radiother Oncol. 2018;128:182–188. 29459153 10.1016/j.radonc.2018.01.019

[18] Heerkens H , Reerink O , Intven MPW , et al. Pancreatic tumor motion reduction by use of a custom abdominal corset. Phys Imag Radiat Oncol. 2017;2:7–10.

[19] Fontana G , Riboldi M , Gianoli C , et al. MRI quantification of pancreas motion as a function of patient setup for particle therapy ‐ a preliminary study. J Appl Clin Med Phys. 2016;17:60–75. 10.1120/jacmp.v17i5.6236 PMC5874090 27685119

[20] Dolde K , Dávid C , Echner G , et al. 4DMRI‐based analysis of inter‐ and intrafractional pancreas motion and deformation with different immobilization devices. Biomed Phys Eng Express. 2019;5:025012.

[21] Wroe AJ , Bush DA , Slater JD . Immobilization consideration for proton radiation therapy. Tech Canc Res Treat. 2014;13:217–226. 10.7785/tcrt.2012.500376 24066953

[22] de Vera P , Abril I , Garcia‐Molina R . Water equivalent properties of materials commonly used in proton dosimetry. Appl Radiat Isot. 2014;83:122–127. 23478093 10.1016/j.apradiso.2013.01.023

[23] Rank C , Heußer T , Buzan MT , et al. 4D respiratory motion‐compensated image reconstruction of free‐breathing radial MR data with very high undersampling. Magn Res Med. 2017;77:1170–1183. 10.1002/mrm.26206 26991911

[24] Möhler C , Russ T , Wohlfahrt P , et al. Experimental verification of stopping‐power prediction from single‐ and dual‐energy computed tomography in biological tissues. Phys Med Biol. 2018;63:025001. 29239855 10.1088/1361-6560/aaa1c9

[25] Batista V , Richter D , Combs SE , Jäkel O . Planning strategies for inter‐fractional robustness in pancreatic patients treated with scanned carbon therapy. Radiat Oncol. 2017;12:94. 28595643 10.1186/s13014-017-0832-x PMC5465513

[26] Stefanowicz S , Stützer K , Zschaeck S , Jakobi A , Troost EGC . Comparison of different treatment planning approaches for intensity‐modulated proton therapy with simultaneous integrated boost for pancreatic cancer. Radiat Oncol. 2018;13:228. 30466468 10.1186/s13014-018-1165-0 PMC6249773

[27] Palmans H , Verhaegen F . Calculated depth dose distributions for proton beams in some low‐ Z materials. Phys Med Biol. 1997;42:1175–1183. 9194136 10.1088/0031-9155/42/6/013

[28] Akbari MR , Yousefnia H . Mirrezai Ehsan. Calculation of water equivalent ratio of several dosimetric materials in proton therapy using FLUKA code and SRIM program. Appl Rad Iso. 2014;90:89–93. 10.1016/j.apradiso.2014.03.012 24705011

[29] Paganetti H . Range uncertainties in proton therapy and the role of Monte Carlo simulations. Phys Med Biol. 2012; 57:R99–R117. 22571913 10.1088/0031-9155/57/11/R99 PMC3374500

[30] van der Horst A , Wognum S , Dávila Fajardo R , et al. Interfractional position variation of pancreatic tumors quantified using intratumoral fiducial markers and daily cone beam computed tomography. Int J Radiat Oncol Biol Phys. 2013;87:202–208. 23790774 10.1016/j.ijrobp.2013.05.001

[31] Naumann P , Habermehl D , Welzel T , Debus J , Combs SE . Outcome after neoadjuvant chemoradiation and correlation with nutritional status in patients with locally advanced pancreatic cancer. Strahlenther Onkol. 2013;189:745–752. 23896631 10.1007/s00066-013-0393-3

[32] Lin L , Souris K , Kang M , et al. Evaluation of motion mitigation using abdominal compression in the clinical implementation of pencil beam scanning proton therapy of liver tumors. Med Phys. 2016;44:703–712. 10.1002/mp.12040 28133755

